# An innovative strategy for control of fungus gnats using entomopathogenic nematodes alone or in combination with waterlogging

**DOI:** 10.21307/jofnem-2020-057

**Published:** 2020-07-06

**Authors:** Chaoying Chen, Haikun Ma, Mingyang Ma, Jingjing Li, Shuyuan Zheng, Qifeng Song, Xinghui Gu, David Shapiro-Ilan, Weibin Ruan

**Affiliations:** 1 College of Life Sciences, Nankai University, Tianjin 30071, China; 2 Yuxi Tobacco Company, Yunnan, 653100, China; 3 USDA-ARS, SEFTNRL, Byron, GA 31008

**Keywords:** *Allium tuberosum*, *Bradysia odoriphaga*, *Heterorhabditis bacteriophora*, *Steinernema feltiae*, Waterlogging

## Abstract

Chive gnat (*Bradysia odoriphaga*) is a soil-borne pest of Chinese chives, which causes millions of dollars in yield losses per year. Traditional methods, such as chemical pesticides leave detrimental chemical residues on plants, which potentially threaten human health. To find a sustainable method of reducing the chive gnat, the authors evaluated the effects of waterlogging and the addition of entomopathogenic nematode (EPN) on reducing chive gnat in Chinese chives via three pot experiments and one field demonstration. Results indicated that increasing the duration of waterlogging markedly increases chive gnat mortality. The presence of EPN also caused chive gnat mortality to increase with exposure time. Most importantly, the combination of waterlogging and EPN had synergistic effects on chive gnat mortality; the combination led to higher mortality than using waterlogging and EPN alone. The study demonstrated that a combination of two environmental friendly methods of fungus gnat control could lead to synergistic effects, which may provide novel approaches to economic and environmentally sustainable pest management measures.

Fungus gnats (*Bradysia* spp., Sciaridae) are small, dark-colored flies whose larval stages feed on organic matter and fungi. They are also opportunistic herbivores on plant roots and underground stem tissues, causing significant yield losses in crops (Arimoto et al., 2018; Katumanyane et al., 2018a, b; Gou et al., 2019).

The chive gnat, *Bradysia odoriphaga* Yang and Zhang, feeds on more than 30 species plants of at least seven families (Zhang et al., 2016). On Chinese chive (*Allium tuberosum* Rottler ex Sprengel), an important perennial herbaceous cash vegetable in eastern Asia, *B. odoriphaga* causes 30 to 80% yield losses, and in severe cases, the crop must be replanted (Ma, Chen, Moens, Han, and De Clercq, 2013). The larval stages of *B. odoriphaga* are cryptic in the soil, and therefore excessively toxic pesticides have been used by farmers leading to pesticide contamination (Yang et al., 2010; Watanabe and Miyake, 2018). Pesticide applications are required after harvest, before a production cycle. The risk of human poisoning is considerable due to the relatively short period between harvest and pesticide application (15-20 d). In addition, overuse of pesticides has also led to the development of pesticide resistance in *B. odoriphaga*, even for neonicotinoids (Chen et al., 2017, 2019).

To reduce the need for reliance on chemical pesticides, many environmentally-friendly approaches have been used against *Bradysia* spp., including abiotic approaches, such as sticky plates to trap adults, ozone water (Shi et al., 2016), soil solarization (Shi et al., 2018), and biotic approaches, such as *Beauveria bassiana* (Hugo et al., 2018), entomopathogenic nematodes (EPNs) (San-Blas et al., 2017), etc. Among them, EPNs have been intensively studied and considered to be potential alternative control agents for *Bradysia* spp. on a large scale (San-Blas et al., 2017; Katumanyane et al., 2018b).

EPNs of the genera *Steinernema* and *Heterorhabditis* (Rhabditida: Steinernematidae and Heterorhabditidae) have been successfully used to control various soil-dwelling and wood-boring insect pests, i.e. *B. odoriphaga* (Ma, Chen, Moens, De Clercq, Li and Han, 2013; Shapiro-Ilan et al., 2018). *Steinernema feltiae* and *H. bacteriophora* isolates collected from diverse location in Northern China showed high virulence to late instar *B. odoriphaga* (Ma, Chen, Moens, De Clercq, Li and Han, 2013). A pot study revealed that higher mortality of *B. odoriphaga* was caused by *H. bacteriophora* than *S. carpocapsae*, and higher mortality of *B. odoriphaga* was obtained when EPNs were applied as cadavers compared to aqueous application (Bai et al., 2016). Two EPN species collected from Northern China, *S. feltiae* JY-17 and *S. hebeiense* JY-82 significantly reduced the larval population of *B. odoriphaga* in a chive field at a level similar to the pesticide phoxim; the average population of *B. odoriphaga* was 3.8, 4.4, 3.9, and 9.6 larvae/stem in the Phoxim, *S. feltiae* JY-17, *S. hebeiense* JY-82 and control plots, respectively (Ma, Chen, Moens, Han and De Clercq, 2013). In another field study, the population of *B. odoriphaga* was reduced by approximately 50% by *S. feltiae* (SF-SN) (Wu et al., 2017). Although EPNs have shown promise to control *B. odoriphaga*, their efficacy in the field generally does not meet grower demands, and thus the potential for EPN use as a stand-alone treatment is reduced.

Taking various measures to improve efficacy is a crucial step to control subterranean insect pest via EPNs (Labaude and Griffin, 2018; Oliveira-Hofman et al., 2019). To improve the efficiency of EPN against target insects, research has explored combining EPNs with entomopathogenic fungi (Mc Namara et al., 2018), bacteria (Jaffuel et al., 2019), or even certain chemical insecticides (Viteri et al., 2018; Yan et al., 2019). However, the potential effect of combining EPN with abiotic approaches has rarely been reported.

It was documented that short-term flooding has been shown to successfully control soil insect pests such as the cranberry girdler, *Chrysoteuchia topiaria* and wireworm, *Agriotes obscurus*, several grasshopper species, the root weevil *Diaprepes abbreviates* (Martin et al., 2010) and citrus fly, *Bactrocera minax* (Brust et al., 2007; Fitzpatrick, 2007; Li et al., 2019; van Herk and Vernon, 2006; Woodman, 2015). For instance, *Diabrotica* larvae are susceptible to flood-induced mortality (Hoback et al., 2002). Moisture content reached a saturation point and caused serious mortality in pupae of *Ceratitis capitata* (Eskafi and Fernandez, 1990). Previous investigations indicated there was a tendency toward decreased pupation rate and egg hatching of *B. odoriphaga* under high soil moisture (≥25%) (Cao, 1985). Currently, it remains unknown how waterlogging directly impacts fungus gnat larvae. However, it was documented that fungus gnat larvae were found to be relatively tolerant to hypoxic conditions upon exposure for 12 to 18 hr to >99% N_2_ or CO_2_ gas, which caused complete mortality of aphids, mites, thrips, and whiteflies but still had 30% survival of fungus gnat larvae (Held et al., 2001). Nonetheless, we propose that waterlogging might also lead to low viability of fungus gnat larvae. Moreover, EPN infectivity increases with soil moisture content, indicating that EPN application under favorable soil moisture levels is critical for EPN efficacy (Koppenhöfer and Fuzy, 2007). Therefore, we hypothesized that the combination of flooding and EPN might exert synergistic effects against *B. odoriphaga* larvae relative to application of EPNs alone.

The objectives of the present study were: to assess the flooding effect on the mortality of *B. odoriphaga* larvae; to compare the effects of different EPN species against *B. odoriphaga* larvae; and to determine whether EPN in combination with flooding has a synergistic effect on controlling *B. odoriphaga* larvae.

## Materials and methods

### Insects, nematodes, and chive preparation

#### Insects

The larvae of *B. odoriphaga* Yang and Zhang were collected from the green chive experimental base located in Wuqing District, Tianjin, China. The larvae were placed on the filter paper (1.5% agar on the filter paper). In a Petri dish (9 cm), fresh chive bulbs were added as feed, and Petri dishes were placed in a growth incubator at 25°C ± 1°C.

#### Entomopathogenic nematodes

The following EPNs were used in this study: four exotic strains, *Steinernema feltiae* (SN strain, France), *S. carpocapsae* (All strain, Georgia, USA), *Heterorhabditis bacteriophora* (HB1 strain, Australia), *H. indica* (provided by USDA-ARS Byron, Georgia USA), as well as two indigenous strains *S. gongdongenese* 74-1 and *H. bacteriphora* 68-1 isolated from Yunnan Province, China by the Nematology lab, Nankai University, China. All strains were reared in *Galleria mellonella* (Woodring and Kaya, 1988). Infective juveniles (IJs) were collected on White traps at least 5 to 7 d after the start of emergence from *G. mellonella* cadavers (White, 1927). IJs were rinsed with sterile water three times, and stored at 14˚C and used within 3 wks.

#### Test plants

One-year old *Allium tuberosum* Rottler ex Sprengel plants (cultivar: Jiuxing 18) were collected from a field with a high incidence of *B. odoriphaga* located at Baodi, Tianjin, China (117.21755 E, 39.739682 N). The average density of *B. odoriphaga* larvae reached 241 larvae per colony (*n* = 8) based on a preliminary survey via counting the number of larvae inside the area of stem and root tissue from 8 randomly selected colonies.

### Experiment 1: waterlogging effects on larval mortality of *B. odoriphaga*

In total, 28 chive plants of similar height and with confirmed infestations of *B. odoriphaga* larvae were selected. The aboveground parts of the plants were removed. Each underground part was placed in a plastic bowl (diameter 10 cm). Approximately 200 ml of sterile water was added to immerse the roots of the chive. The experiment was consisted of six treatments with different waterlogging periods: 10 min, 20 min, 40 min, 1 hr, 2 hr, and 4 hr. Bowls filled with plants without water addition were used as control (0 min). The plastic bowl was sealed with Parafilm to prevent larvae from escaping as well as maintaining moisture. Larval mortality in each bowl was recorded immediately after the corresponding waterlogging period was completed. All these bowls were kept at room condition at 23 to 25˚C and 25 to 30% relative humidity. There were four replicates for each treatment. The experiment was conducted twice in time.

### Experiment 2: the virulence of six EPN isolates against the *B. odoriphaga* larvae

Ten 4th instar larvae of *B. odoriphaga* were randomly chosen from the infested chive plants and placed on the bottom of a Petri dish (3.5-cm diameter) lined with filter paper. After that 150 μl nematode suspension containing approximately 1,000 IJs was added to the Petri dish, and the same volume of sterile water was added to control dishes. Petri dishes were sealed with Parafilm to maintain moisture and placed into an incubator at 23 ± 1°C in dark. Larval mortality was recorded at 24 hr intervals. The dead larvae were dissected under a stereomicroscope to confirm that the mortality was from EPN infection. The experiment consisted of the six EPN isolates indicated above plus a non-treated control. Each treatment and the control had eight replications. The experiment was conducted twice in time.

## Experiment 3: the effects of the EPN and waterlogging alone or in combination

### Pot trial

Chive plants of similar height were selected. The leaves were cut close to the base of the stem, and the remains of chive plants were buried in a new plastic bowl (10 cm in diameter) filled with moistened vermiculite (vermiculite: sterile water = 1:2, V/V) containing 50 mL vermiculite and 100 mL sterile water. The experiment consisted of five treatments: waterlogging for 4 hr; waterlogging for 2 hr; waterlogging for 2 hr and subsequent addition of EPN; EPN addition alone; and no waterlogging or EPN addition as a control. Based on results of Experiment 2, *H. bacteriophora* and *S. feltiae* was selected for this experiment. A total of 1 ml nematode suspension (20 IJ/μl) was added near the bulb of the chive plant in the EPN treatments. For the combination treatment, the plants were first exposed to waterlogging, then water was poured off and EPNs were added. Based on the results of Experiment 2, larval mortality was assessed 96 hr after EPN application. Each treatment had five replications. The entire experiment was conducted twice in time.

### Field trial

On March 29, 2019, the field experiment was set up, located at Diliubu County, Xiqing District, Tianjin, China. Corn was planted in the field in the previous growing season. Before initiation of the experiment, the soil was carefully checked and no *B. odoriphaga* larvae were detected. The field experiment consisted of six blocks. In each block, five 30 cm × 30 cm rectangular plots were randomly selected as the experimental treatment plots. The treatments were: waterlogging for 4 hr; waterlogging for 2 hr; waterlogging for 2 hr with EPN addition; EPN addition alone; and without waterlogging and EPN addition (control), in total 30 plots. The plots were at least 2 m apart. To conduct the waterlogging treatment, four wooden sticks were inserted at the four corners of the plot with 30 cm height above the ground, and then the inside of each plot was surrounded by 20 cm height plastic paper, with 10 cm below the ground and 10 cm above the ground. In each plot, 100 individual chive plants were transplanted. Plants were subsamples of chive plants collected from the field, and heavily infested with *B. odoriphaga* larvae as described before. Tap water was consecutively added to the corresponding waterlogging plot to completely immerse the plot for 2 or 4 hr. For the rest of treatments (EPN alone or control treatments), the soil was thoroughly watered by tap water following standard grower’s practice. Once the waterlogging process was completed, nematode suspensions (100,000 IJs in 10 mL per plot) were evenly added to the corresponding plots. For the non-EPN treatment, equal volume of sterile water was added. After the EPN addition and waterlogging process, a 60-mesh nylon net was placed over each plot, drawn to the ground and sealed around with the soil, preventing other insects from entering. The experiment was set up in the late afternoon to avoid EPNs exposure to strong UV light. Based on the results of pot trial in Experiment 3, *H. bacteriophora* was chosen for the field trial. After 60 days, chives were harvested on May 28, 2019, and the seedlings were cut close to the soil surface and oven-heated at 80˚C to determine dry weight for each treatment.

### Statistical analysis

All experiments except field trial were conducted twice, and the data were combined based on a lack of statistical differences between trials. Before conducting analyses, data were checked for homogeneity of variance and normality was confirmed by inspection of the residuals.

One-way or two-way ANOVAs were implemented to test treatment effects in Experiments 1 and 3 (pot test and field trial), respectively. In Experiment 2, the effects of six EPN species and different observation times on the number of dead larvae of *B. odoriphaga* were determined using a repeated measure ANOVA, with EPN species used as main factor, and observation time as error structure. In all experiments, Tukey’s test was used to further elucidate treatment differences. All the analyses were done in R software version 3.5.1 (2018-07-02) (R Core Team, 2018).

## Results

### Experiment 1: waterlogging effects on larval mortality of *B. odoriphaga*

**Figure 1:**
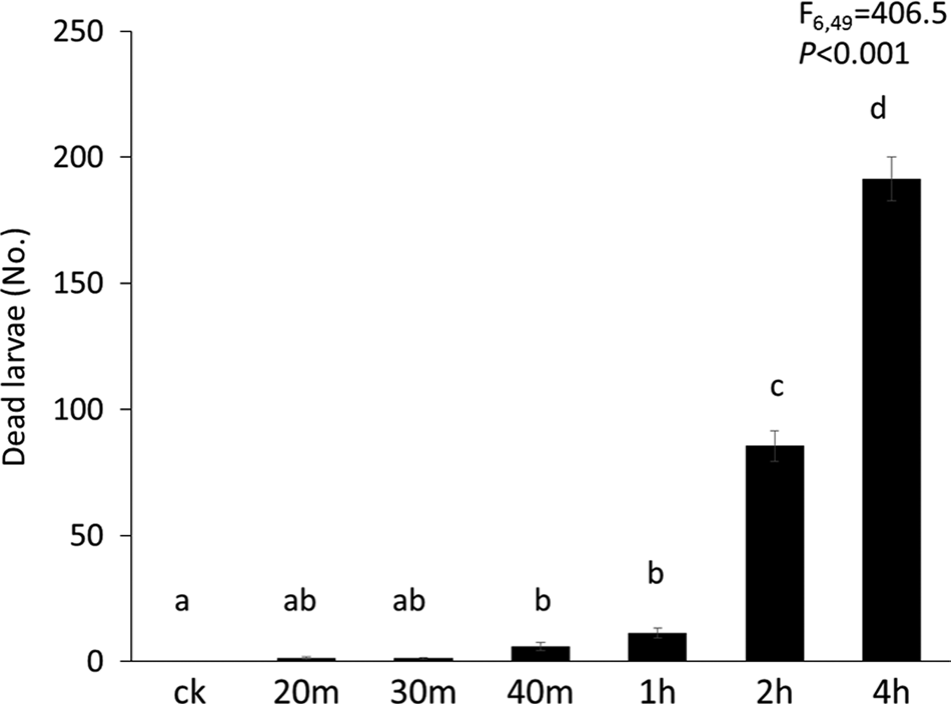
The effects of six waterlogging time intervals on *Bradysia odoriphaga* mortality. ck: no waterlogging treatment, 20 min, 30 min, 40 min, 1 hr, 2 hr, 4 hr: waterlogging time. Statistics presented in the upper part of the figure represent results from a one-way ANOVA. Bars represent the number of dead larvae of total (241 on average) in each colony of chive. Bars with identical letters are not significantly different based on a Tukey *post hoc* test (P < 0.05).

Larval mortality of *B. odoriphaga* increased significantly with increased waterlogging time (df = 6,49, *F* = 406.5, *P* < 0.001, Fig. 1). Waterlogging for 2 and 4 hr significantly increased larval mortality compared with waterlogging for 1 hr and less. Waterlogging the chive for 4 hr doubled *B. odoriphaga* mortality compared with waterlogging for 2 hr (Fig. 1).

### Experiment 2: the virulence of six EPN isolates against the *B. odoriphaga*

**Figure 2:**
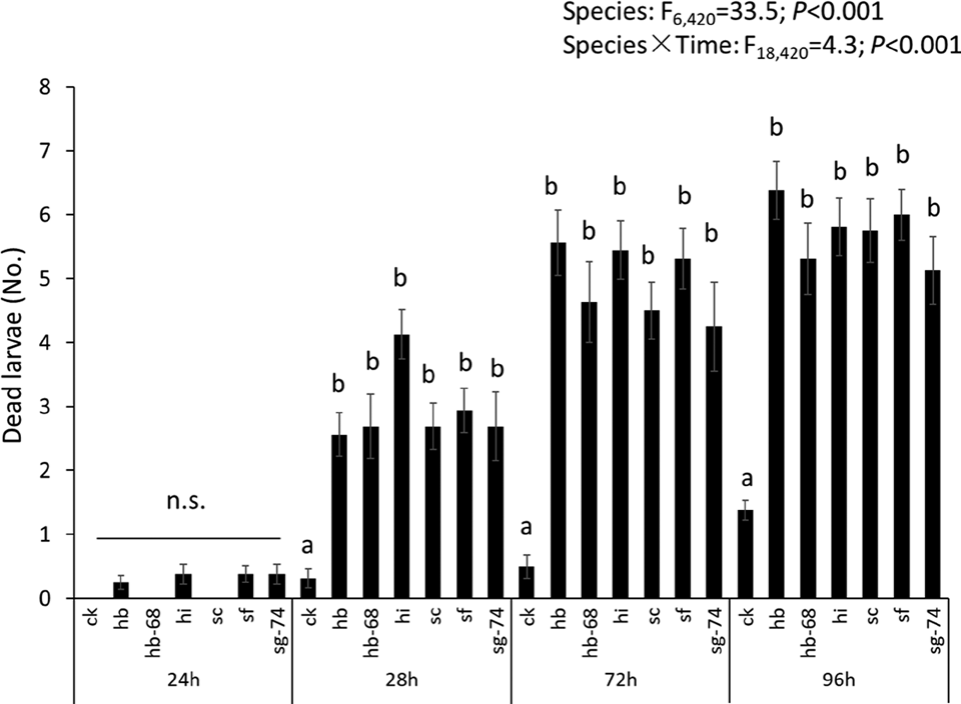
The effects of six entomopathogenic nematode strains and four waterlogging times on *Bradysia odoriphaga* mortality. 24 hr, 48 hr, 72 hr, 96 hr: exposure time to entomopathogenic nematodes. ck: no entomopathogenic nematodeaddition; hb: *Heterorhabditis bacteriophora*; hb-68: *H. bacteriophora*-68; hi: *H. indica*; sc: *Steinernema carpocapsae*; sf: *S. feltiae*; sg-74: *Steinernema* sp. Statistics presented in the upper part of the figure represent results from a repeated measure ANOVA. Bars represent the number of dead larvae out of 10 total. n.s.: no significant differences. Bars with identical letters within waterlogging time are not significantly different based on a Tukey *post hoc* test (*P* < 0.05).

Overall, EPN caused higher mortality of *B. odoriphaga* relative to the control without EPN addition regardless of EPN species (df = 6,420, *F* = 33.5, *P* < 0.001, Fig. 2). The mortality of *B. odoriphaga* increased with exposure time of EPN. In particularly, high mortalities were caused by *H. bacteriophora*, *H. indica*, and *S. feltiae* at 72 hr and *H. bacteriophora* and *S. feltiae* at 96 hr (Fig. 2).

### Experiment 3: the effects of the EPN and waterlogging alone or in combination

**Figure 3:**
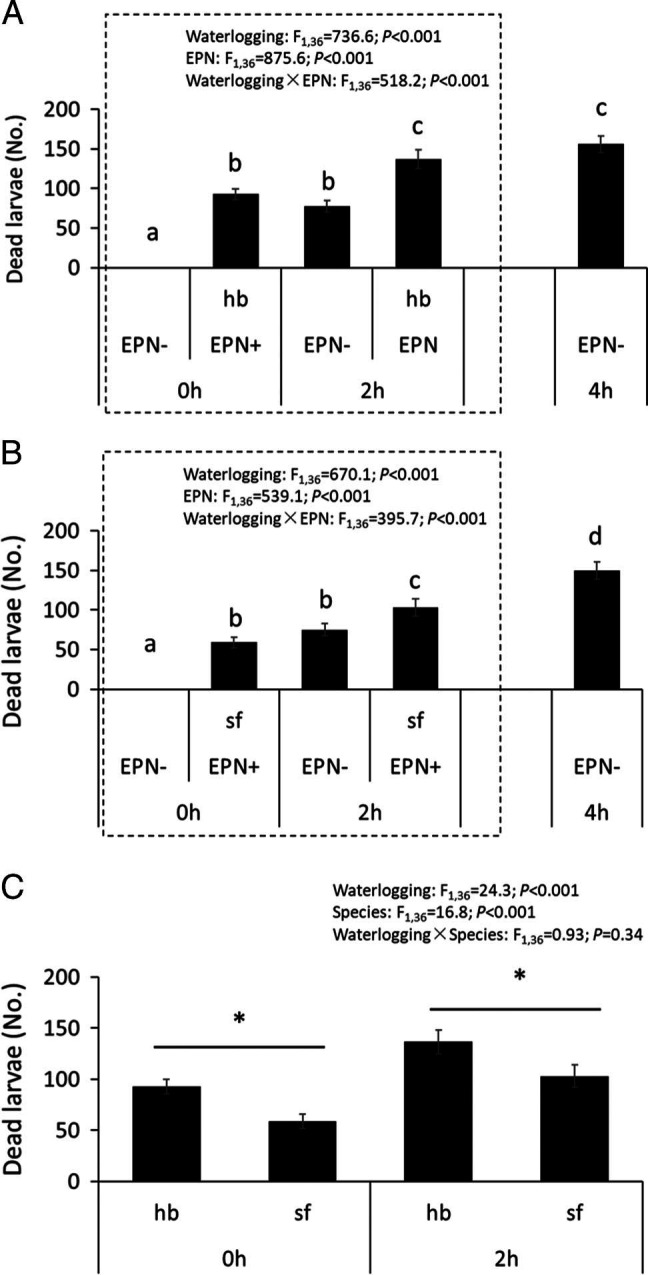
Mortality of *Bradysia odoriphaga* following entomopathogenic nematode addition and waterlogging. (A): *H. bacteriophora* and waterlogging treatment, (B): *S. feltiae* and waterlogging treatment, (C) both entomopathogenic nematode species (EPN) and waterlogging treatment (C). hb: *Heterorhabditis bacteriophora*; sf: *Steinernema feltiae*. 0 hr, 2 hr, 4 hr: no waterlogging, waterlogging for 2, 4 hr, respectively. “EPN+, EPN−” indicates with and without EPN addition, respectively. Bars represent the number of dead larvae of total (241 on average) in each colony of chive. Statistics presented in the upper part of the figure are following a two-way ANOVA. “*” in Fig. 3C indicates significant differences between two EPN species in within that waterlogging treatment. Different letters above set of bars indicate significant difference between EPN and waterlogging treatment (*P* < 0.05).

Either waterlogging or adding EPN alone significantly increased *B. odoriphaga* mortality compared with the control treatment (Fig. 3). For both *H. bacteriophora* and *S. feltiae*, waterlogging for 2 hr significantly increased *B. odoriphaga* mortality compared with only adding EPN species alone (df = 1,36, *F* = 726.6, *P* < 0.001, Fig. 3A; df = 1,36, *F* = 670.1, *P* < 0.001, Fig. 3B). Waterlogging for 4 hr led to the highest death rate of *B. odoriphaga* compared with all other treatments in Fig. 3B with *S. feltiae*. Further, when *H. bacteriophora* and *S. feltiae* were compared, *H. bacteriophora* showed stronger effects on reducing the number of *B. odoriphaga* than *S. feltiae* with and without waterlogging (df = 1,36, *F* = 24.3, *P* < 0.001, Fig. 3C).

**Figure 4:**
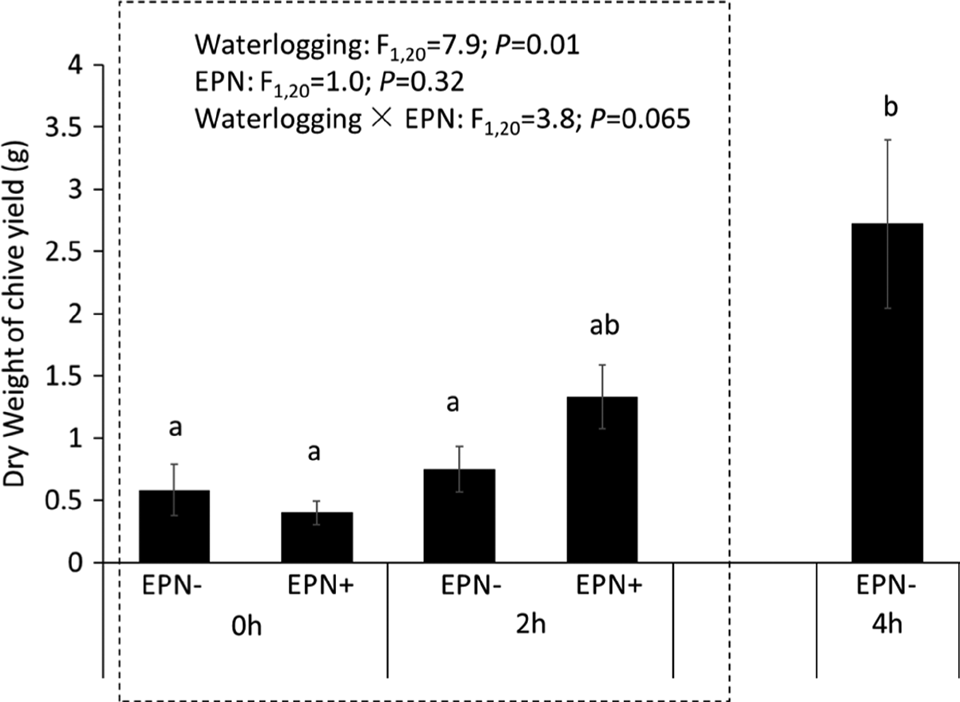
The effects of entomopathogenic nematodes and waterlogging on the dry weight of chive. 0 hr, 2 hr, 4 hr: no waterlogging, waterlogging for 2, 4 hr, respectively. “EPN+, EPN−” indicates with and without the addition of entomopathogenic nematode (EPN), respectively. Statistics presented in the upper part of the figure indicate results from a two-way ANOVA. Different letters above bars indicate significant difference among treatments (*P* < 0.05).

With regard to field experiment, based on the results of two-way ANOVA with two main factors (EPN+/−, 2 hr-waerloging+/−), waterlogging had significant effects on the plant growth (df = 1,20, *F* = 7.96, *P* = 0.01, Fig. 4). There was a marginary interaction effect of waterlogging and EPN (df = 1,20, *F* = 3.82, *P* = 0.065, Fig. 4). Four hours of waterlogging sustained significantly higher dry weight of chive relative to other treatments (Fig. 4).

## Discussion

Our study, based on results of the plate and pot experiments, shows that EPN application and waterlogging both had negative effects on *B. odoriphaga* populations. Longer waterlogging times led to higher death of *B. odoriphaga*, and all EPN species caused mortality in *B. odoriphaga* larvae. Most importantly, waterlogging and EPN addition interacted positively on *B. odoriphaga* mortality. The combined treatments of EPNs and waterlogging caused higher mortality in *B. odoriphaga* larvae than either of the treatments alone.

The relationship between the moisture and fungus gnat *Bradysia* sp. nr. *coprophila* mortality was also reported in a previous study, in which the authors found a moisture content between 30 and 68% was optimum for fungus gnat survival (Cloyd and Dickinson, 2008), and when the moisture content exceeded 68%, the survival rate of *Bradysia* sp. nr. *coprophila* would decrease. Other pests, such as, *Diabrotica* larvae are susceptible to flood-induced mortality (Hoback et al., 2002). Soil saturation (at −0.01 bars) induced mortality in pecan weevil, *Curculio caryae* (Shapiro-Ilan et al., 2006). Under exposure to severely hypoxic water, the pupae of semi-terrestrial caddisfly were most sensitive relative to aquatic larvae and terrestrial larvae (Cavallaro and Hoback, 2014). Increasing the duration of the larval immersion of *Bactrocera minax* in water also led to the increased mortality (Li et al., 2019). This study is the first report on the effects of the waterlogging approach on the genus *Bradysia*. In the case of *B. odoriphaga*, further studies are still needed to elucidate the relationship between larval mortality and the concentration of available oxygen during the waterlogging process.

We tested the effect of EPN on *B. odoriphaga* larvae including four exotic species and two indigenous isolates. The results indicated that all EPN species significantly reduced the number of *B. odoriphaga* larvae compared with no EPN addition. This is consistent with Ma, Chen, Moens, De Clercq, Li and Han (2013) which also found that EPN species had great potential to control *B. odoriphaga*. Higher virulence was observed in *H. bacteriophora* relative to *S. feltiae* in the present study, and this is consistent with the results of Bai’s findings (Bai et al., 2016).

We found that compared to EPN and waterlogging applied alone, their combination caused higher mortality in *B. odoriphaga* larvae. Waterlogging caused a high level of soil moisture that might facilitate EPN movement and penetration. Unfavorable conditions may result in a low efficacy of EPN and impede field application on a large scale; particularly low moisture tends to be detrimental. In another study, nematode virulence to the insect pest increased with soil moisture content for all EPN species and isolates tested, even near the saturation point (Grant and Villani, 2003), indicating low moisture is a key limiting factor restricting EPN efficiency under field conditions. Shapiro-Ilan et al. (2006) also observed higher EPN induced mortality of the target pest (*C. caryae*) in progressively higher soil moisture contents, yet in this case levels near saturation for ten days decimated the pest population such that any additional mortality from the EPNs was negligible. In addition, we proposed that waterlogging might impair the fungus gnat’s immunity and thus fungus gnats might be more vulnerable to attack by EPNs. This hypothesis remains to be investigated.

In our field trial, waterlogging or EPN application (or their combination) did not induce a significant effect on chive yield. Generally, it may be harder to show yield differences instead of direct effects on insect mortality (as observed in the other experiments). Four hours of waterlogging had the highest biomass yield in the field trial, which is in consistent with the highest *B. odoriphaga* mortality in the pot trial. This is in agreement with the findings of Steffan et al. (2012) who also used water flooding techniques to effectively replace the corresponding pesticide applications and control the most economically important pests in cranberries. However, previous studies that tested flood duration effects on pest control in cranberries found that too short a flood duration would lead to inadequate control against the pest; thus, the waterlog duration might be extremely crucial for insect control (Fitzpatrick, 2007). On the other hand, we know that most plant species cannot survive prolonged soil waterlogging. Thus, we did not continue to explore flooding for a longer period of time than 4 hr-waterlogging in combination with EPN treatments. Future studies are needed to test the effects of waterlogging for longer-periods on *B. odoriphaga* larvae and how this influences the chive plant mechanistically.

Dissolved oxygen content might be correlated with the minimum time needed to kill *B. odoriphaga* larvae. We hypothesize that the time used for killing the larvae might be shorter if using hypoxic water such as groundwater compared with stream water since the latter has a high amount of dissolved oxygen. It was reported that drip irrigation systems can cause the recovery of dissolved oxygen from hypoxic water (Maestre-Valero and Martinez-Alvarez, 2010). Thus, to control *B. odoriphaga* larvae, surface irrigation might be better than drip irrigation. Further studies are needed to elucidate these hypotheses. Conceivably, environmentally friendly substances to exhaust the dissolved oxygen in the water could be added, thereby shortening the time to kill *B. odoriphaga* larvae. However, the extent that these approaches might cause the negative effect on the EPNs or other beneficial organisms in the soil also needs further study.

## Conclusion

In contrast to the current serious problem caused by overusing chemical pesticides to control chive gnat, this study indicated that EPN, waterlogging alone and the combination of EPN and waterlogging could be good biological approaches to controlling chive gnat. These novel findings have substantial potential for establishing a sustainable and environmental-friendly pest management program for chive gnat as well as other fungus gnats.
